# The Antiproliferative and Apoptotic Effects of Capsaicin on an Oral Squamous Cancer Cell Line of Asian Origin, ORL-48

**DOI:** 10.3390/medicina55070322

**Published:** 2019-06-28

**Authors:** Mohammad Firdaus Kamaruddin, Mohammad Zakir Hossain, Aied Mohamed Alabsi, Marina Mohd Bakri

**Affiliations:** 1 Department of Oral and Craniofacial Sciences, Faculty of Dentistry, University of Malaya, Kuala Lumpur 50603, Malaysia; 2 Department of Oral Physiology, Faculty of Dentistry, Matsumoto Dental University, Shiojiri, Nagano 399-0781, Japan; 3 Department of Oral Biology and Biomedical Sciences, Faculty of Dentistry, Mahsa University, Jenjarom 42610, Selangor, Malaysia

**Keywords:** capsaicin, cancer, oral, apoptosis, antitumor, antiproliferative

## Abstract

*Background and Objectives*: The antitumor activities of capsaicin on various types of cancer cell lines have been reported but the effect of capsaicin on oral cancer, which is prevalent among Asians, are very limited. Thus, this study aimed to investigate the effects of capsaicin on ORL-48, an oral cancer cell line of Asian origin. *Materials and Methods*: Morphological changes of the ORL-48 cells treated with capsaicin were analyzed using fluorescence microscopy. The apoptotic-inducing activity of capsaicin was further confirmed by Annexin V-Fluorescein isothiocyanate / Propidium iodide (V-FITC/PI) staining using flow cytometry. In order to establish the pathway of apoptosis triggered by the compound on ORL-48 cells, caspase activity was determined and the mitochondrial pathway was verified by mitochondrial membrane potential (MMP) assay. Cell cycle analysis was also performed to identify the cell cycle phase of ORL-48 cells being inhibited by the capsaicin compound. *Results*: Fluorescence microscopy exhibited the presence of apoptotic features in capsaicin-treated ORL-48 cells. Apoptosis of capsaicin-treated ORL-48 cells revealed disruption of the mitochondrial-membrane potential, activation of caspase-3, -7 and -9 through an intrinsic apoptotic pathway and subsequently, apoptotic DNA fragmentation. The cell cycle arrest occurred in the G1-phase, confirming antiproliferative effect of capsaicin in a time-dependent manner. *Conclusion*: This study demonstrated that capsaicin is cytotoxic against ORL-48 cells and induces apoptosis in ORL-48 cells possibly through mitochondria mediated intrinsic pathway resulting in cell cycle arrest.

## 1. Introduction

Oral squamous cell carcinoma (OSCC) is the sixth most common cancer with an estimated prevalence of 400,000 new cases and about 211,000 deaths recorded annually [1]. Among the main etiological factors associated with cancers are tobacco use, ethanol consumption and microbial infection [2,3,4]. There has been little advancement in the death rate of oral cancer patients especially in the late stages of the disease where the lymph nodes may be involved causing further metastasis of the disease and impingement upon critical structures [5]. There is a wide variation in the geographical distribution of oral cancer and that several countries with the highest incidence of oral cancer in the world are located in South Asia where India has often been cited as the country with the highest incidence of oral cancer in the world [6]. New cases of oral cancer are anticipated each year, and the countries with the highest rates are Sri Lanka, India, Pakistan, Bangladesh, Hungary and France [7]. Among the various types of methods used to treat cancer include surgery, radiation therapy and chemotherapy. Among the challenges associated with chemotherapy treatment is the occurrence of toxicity on the remaining healthy cells and although the present chemotherapeutic drugs improved the overall survival rate of the patients, the outcome of treatment remains poor due to the development of drug resistance and severity of the side effects [8]. Thus, alternative treatments such as the use of plant based antitumor agents have also generated a lot of interest. Historically, natural products have been utilized since ancient times for the treatment of many diseases and illnesses [9]. Herbal medicines have a wide range of pharmaceutical potential and served as an important novel source for the treatment of a variety of human ailments including cancer [10]. A report by World Health Organization (WHO) stated that an increase consumption of natural product can assist in the modulation of key signalling cascades that will induce apoptosis and inhibit cell proliferation resulting in prevention of one-third of all cancer deaths [11]. 

Capsaicinoids are a group of chemical compounds found in chili and the main capsaicinoid in chili is capsaicin. Pharmacological and pain research studies have shown multiple effects of capsaicin in a variety of physiological systems including pain, cardiovascular, digestive and urinary systems [12,13,14,15,16]. It has analgesic and anti-inflammatory activities and topical creams/gels with capsaicin are used to treat chronic pain, including cancer related and neurogenic pain [12,13,14,15,16]. Capsaicin, an active compound from plants belonging to the genus. Various studies have reported that capsaicin has antiproliferative effects towards cancer cells mainly through programmed cell death induction, as well as in the regulation of transcription factor expression, growth signal transduction pathways and cell cycle progression arrest [17,18,19]. Due to its ability to mediate cell cycle arrest and induce cell apoptosis, capsaicin has been shown to be cytotoxic in many different types of cancer cells including breast cancer, colon cancer, hepatocarcinoma and non-small cell lung cancer [20,21,22,23]. Despite the accumulating studies of antitumor effects of capsaicin, there have been very few studies on the effects of capsaicin on oral cancer cell lines. Thus, this current study serves to determine the antitumor effects of capsaicin on an Asian oral cancer cell line, ORL-48.

## 2. Materials and Methods

### 2.1. Cell Culture

The human OSCC cell line, ORL-48 [24], was purchased from Cancer Research Malaysia and Foundation, Subang Jaya Medical Center (CARIF, Selangor, Malaysia). Cells were cultured in Dulbecco’s modified Eagle’s medium (DMEM) F-12 medium supplemented with 10% (*v*/*v*) foetal bovine serum and antibiotic solution of 2 mL of penicillin-streptomycin with 1 mL of amphotericin B.

### 2.2. Capsaicin Compound Preparation

The active compound Capsaicin (Geneion Bio, Malaysia) was prepared according to the manufacturer’s protocol; 3.1 mg of capsaicin dissolved in 1 mL 100% DMSO (dimethyl sulfoxide) to make a 10 mM capsaicin stock solution and kept in 4 °C before being diluted into the desired concentrations of 50, 100, 150, 200, 250, 300 and 350 μM using DMEM-F12. 

### 2.3. 3-(4,5-Dimethylthiazol-2-yl)-2,5-Diphenyltetrazolium Bromide (MTT) Assay

Cell viability was measured using 3-(4,5-dimethylthiazol-2-yl)-2,5-diphenyltetrazolium bromide (MTT) assay. Briefly, ORL-48 cell line was cultured and segregated into 96-well plates at a concentration of 1 × 105 cells/mL in a total volume of 100 mL for each well, overnight. After treatment and incubation for 24, 48, 72 h in a humidified 5% CO_2_ incubator at 37 °C, 20 mL of MTT solution (5 mg/mL in phosphate buffer saline; Sigma, St Louis, USA) was added to each well using a repeating pipettor and was mixed gently for one minute on an orbital shaker. The cells were then incubated for 3-4 h at the temperature of 37 °C in a CO_2_ incubator and following this, there would be formation of formazan as dark crystals at the bottom of the wells. The culture medium was then removed and 150 µL DMSO (dimethyl sulfoxide) was then added to each well. The optical density (OD) was then measured based on the optical absorbance read at 575 nm with reference at 650 nm wavelength using an ELISA (ezyme-linked immunosorbent assay) microplate reader (BioTek, Winooski, VT, USA) and analyzed using GEN5 software. The concentration of the culture compound that results in 50% inhibition of the cells population, known as the inhibition concentration (IC50), was represented in a cell viability graph. The assay for each cell line was performed in triplicates in three different experiments. 

To demonstrate the antiproliferative activities of ORL-48 cells induced by capsaicin compound, MTT cell proliferation analysis was carried out. Briefly, around 1 × 105 cells/mL of ORL-48 cells were seeded in a 96-well plate overnight. The cells were then exposed to capsaicin’s IC25, IC50 and IC75 values obtained from the MTT cell viability assay analysis. Following treatment duration of 24, 48 and 72 h, 20 µL of MTT solution (Sigma, St Louis, USA) was added in each well and incubated for 4 h in a humidified 5% CO2 incubator at 37 °C. The supernatant was later removed and 150 µL of DMSO solution was added into each well.

### 2.4. Fluorescent Microscopy

The morphological changes of ORL-48 cells in response to capsaicin were further analyzed under the fluorescent microscope. The ORL-48 cells were seeded in a 6-well plate at a concentration of 3 × 10^5^ cells/mL and incubated overnight prior to treatment with capsaicin compound employing the IC_50_ concentration (200 µM) (followed by a separate 24, 48 and 72 h incubation period in the CO_2_ humidified incubator. A group of untreated cells was incubated with DMSO within the same periods and act as the control for the experiment. Following the incubation, harvested cells from the plates were centrifuged at 3000 rpm for 10 min before washing the pellets with phosphate buffer saline (PBS). The cell pellets were then suspended with 5 μL of acridine orange (AO, 10 μg/mL) in addition to 5 μL of propidium iodide, (PI, 10 μg/mL) and incubated for 20 min on ice. A volume of 10 μL of pellet was pipetted on a slide before putting on the cover slip. After 30 min, the double-stained cells were analyzed using fluorescent microscope. The assay was performed in three independent experiments and triplicates for each treatment period.

### 2.5. Flow Cytometric Analysis of Apoptosis by FITC-Annexin V/PI

Fluorochrome-labeled Annexin V was used as a probe to detect exposed translocated membrane phospholipid on the early onset of apoptosis for flow cytometric analysis. Vital dye like propidium iodide (PI) is used in conjunction with Annexin V staining to differentiate the probable apoptotic stages of the ORL-48 cells which were exposed to capsaicin. ORL-48 cells were incubated with capsaicin at a concentration of 200 μM for 24 and 48 h. Following centrifugation, approximately 3 × 10^5^ cells/mL were harvested, washed with cold PBS and then re-suspended in 100 μL of binding buffer. Then 100 μL of the solution were transferred to a culture tube, followed by the addition of 5 μL of FITC-conjugated Annexin V (BD Bioscience-Pharmingen, San Jose, CA, USA) and 5 μL of Propidium iodide PI. The cells were gently vortexed and then incubated for 15 min at room temperature, in the dark. Immediately, 400 μL of binding buffer was added to each tube and the samples were analyzed by flow cytometry FACS Calibur flow cytometer (BD Biosciences, San Jose, CA, USA) to examine the early and late stages of apoptosis. Each sample were analyzed using Cell Quest Pro software (BD Biosciences, San Jose, CA, USA) within 1 h.

### 2.6. Determination of Caspase Activities

Caspase activities of the ORL-48 cells was studied in a downstream analysis using Caspase-Glo assay kit (Promega, Wisconsin, USA). Activation of the different functional group of caspases, apoptotic initiator (caspase-3/7) or apoptotic effector (caspase-8, and-9) was determined as this will provide the knowledge of the source that trigger the apoptosis process. Caspase-3/7, -8 and -9 assays were carried out in triplicates using Caspase-Glo-3/-7, -8 and -9 Kit (Promega, WI, USA) according to the manufacturer’s protocol. Briefly, ORL-48 cells were seeded in 96-well white-walled plate at the concentration of 1 × 10^5^ cells/mL and incubated at 37 °C in 5% CO_2_ incubator overnight. The cells were then treated with capsaicin at its IC_50_ concentration of 200 μM for 24 h and the untreated cells will serve as the negative control. Following the incubation, 100 μL of the Caspase-Glo reagent was added to each well and further incubated at room temperature for 1 h in the dark. Contents of the wells were mixed and the plate was incubated at room temperature for a specific time ranging from 30 min to 3 h, in which the incubation period was determined empirically. Finally, caspases activities were evaluated by measuring the luminescence signal using the uQuant ELISA microplate reader (BioTek, Winooski, VT, USA).

### 2.7. Measurement of Mitochondrial Membrane Potential (Δψ_m_) Assay

Mitochondrial membrane potential (MMP) (Δψ_m_) depolarization of ORL-48 cells in response to capsaicin was assessed using TMRE-MMP Assay kit (Abcam, Cambridge, MA, USA), in triplicate according to the manufacturer’s protocol. ORL-48 cells were seeded in clear bottom, black wall 96-wells plate at 1 × 10^5^ cells/mL and then treated with capsaicin at its IC_50_ concentration of 200 μM for 24 h prior to assessment. TMRE solution was added to the media to a final concentration of 500 nM and incubated for 30 min at 37 °C. The media of each well was then aspirated and the cells were washed with 200 μL of Assay Buffer. The cells were then returned to the incubator for another 15–30 min. Cells were then gently pelleted by centrifugation and the media was removed by aspiration. Cells were then resuspended in 100 μL of 0.2% BSA/PBS and centrifuged again. The cells were resuspended one more time and transferred to a microplate. Fluorescence absorbance was then measured at excitation/emission (Ex/Em) = 549/575 nm using ELISA microplate reader (BioTek, Winooski, VT, USA). The fluorescence intensity for blank reaction was retracted from the experimental value.

### 2.8. Cell Cycle Analysis Using Flow Cytometry

Initially, the cells were seeded into the 6-well plates and incubated overnight in 5% CO_2_ humidified incubator at 37 °C. Subsequently, capsaicin was added in each well at its IC_50_ value for 24, 48 and 72 h. The treated cells were harvested by centrifugation at 500g for 5 min and fixed with ice-cold 70% ethanol, and incubated at 4 °C overnight. Then, the cells were harvested and washed twice with PBS. Next, the cells were stained with PBS containing with 5 μL of FITC-conjugated Annexin V and 5 μL of PI, prior to incubation at room temperature for 30 min in the dark. The cell cycle distribution of the stained cells was assessed using FACS Calibur flow cytometer with Cell Quest Pro software (BD Biosciences, San Jose, CA, USA). The percentage of DNA content distribution in different phases of the cell cycle was analyzed using Modfit LT Software (Verity Software House, Topsham, ME, USA). 

### 2.9. Statistical Analysis

Statistical significance was analysed using one-way analysis of variance (ANOVA) and expressed as mean ± standard error mean (SEM) and student *t*-test.

## 3. Results

### 3.1. MTT Assay

**Figure 1:**
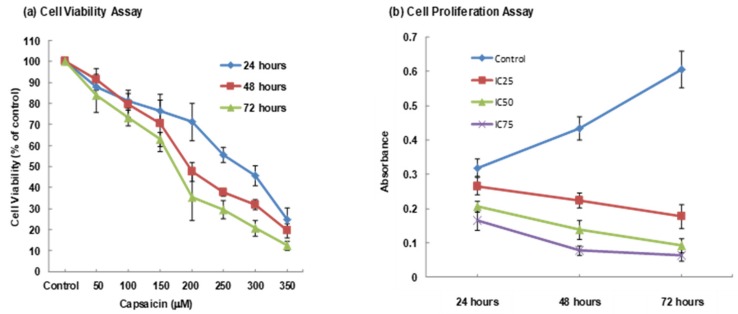
Effect of capsaicin on cell viability (**a**) and proliferation (**b**) of ORL-48 cells. Data are presented as mean ± standard error mean (SEM) from three independent experiments (*n* = 3).

The percentage of viable cells was determined for different concentrations of capsaicin and treatment periods. The cell viability decreased with the time of incubation period in a time-dependent manner (Figure 1a). The inhibitory effect of capsaicin against the cell line was evaluated using the half-maximal inhibition concentration (IC_50_) value of capsaicin (a concentration that is required to inhibit cell growth in 50% of the cell population). The IC_50_ value for capsaicin-treated ORL-48 cell lines was 200 µM at 48 h. Along with increasing concentration of capsaicin, the percentage of cell viability was found to decrease indicating the cytotoxic effect of capsaicin on ORL-48 cells as shown in Figure 1a. 

ORL-48 cell proliferation, when exposed to capsaicin using the IC_25_, IC_50_ and IC_75_ values were determined for 24, 48 and 72 h. Optical density value was measured to determine the proportion of cell proliferation. Figure 1b shows that cells proliferation decreased along with the time of treatment. As opposed to treated cells, untreated cells proliferated along with time of treatment as indicated by the increased in optical density value, shown in Figure 1b. This finding proves the antiproliferation effect of capsaicin on ORL-48 cell line in a concentration and time-dependent manner.

### 3.2. Morphological Analysis

**Figure 2:**
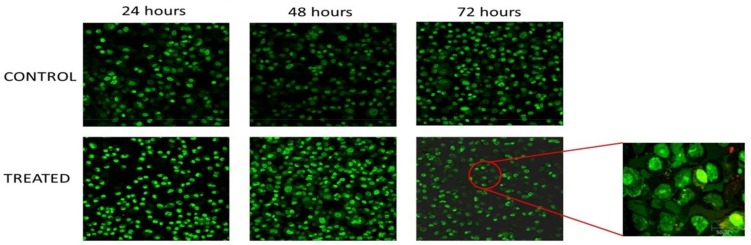
Morphological analysis of capsaicin-treated and untreated (control) ORL-48 cell. Fluorescence microscope images (40×) of 24, 48 and 72 h capsaicin-treated (IC_50_ concentration (200 µM)) and untreated (control) ORL-48 cells with AO/PI double staining, the red circle represents the selected area at 72 h that exhibited morphological changes along with time.

In order to differentiate the apoptotic cells from necrotic cells, the ORL-48 cells were stained with acridine orange (AO) and propidium iodide (PI). As shown in Figure 2, most of the cells of the untreated cell population showed uniform green nuclei (due to high staining of AO) when viewed under the fluorescent microscope. In contrast, capsaicin-treated ORL-48 cells with IC_50_ concentration (200 µM) within 24, 48 and 72 h showed intensified green colored nuclei and apoptotic blebs (Figure 2). Chromatin condensation was also observed in many cells across the treated cell population. It was observed that some cells exhibited orange colored nuclei, in the capsaicin-treated population of the ORL-48 cell line at 72 h as compared to the control cells as shown in Figure 2.

### 3.3. Quantification of Apoptotic/Necrotic Cells by FITC-Annexin V/PI

**Figure 3:**
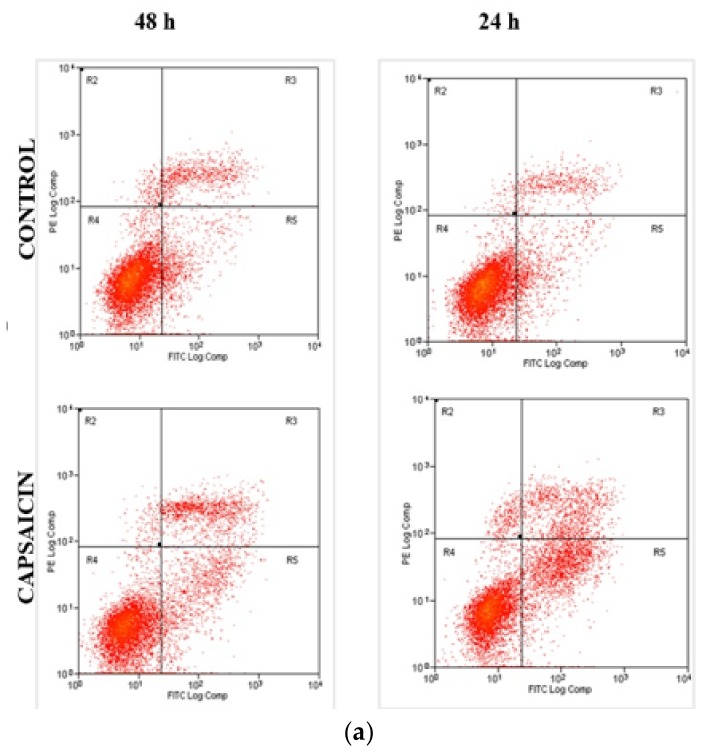

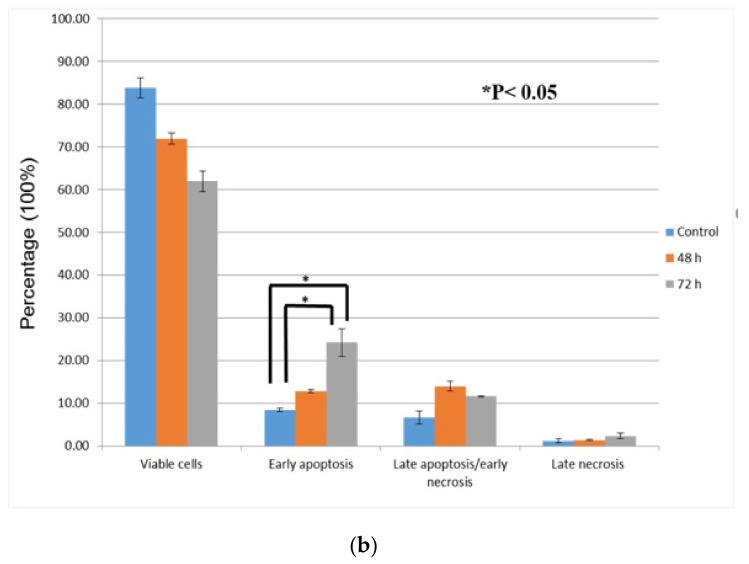
Quantification analysis of Apoptotic/Necrotic cells by Annexin V-Fluorescein isothiocyanate / Propidium iodide (FITC-Annexin V/PI). (**a**) Flow cytometric analysis of apoptosis in ORL-48 cells treated with IC_50_ concentration (200 µM) of capsaicin for 48 h and 72 h using FITC-annexin V/PI double staining. Early and late apoptosis were examined on fluorescence 2 (FL2 for PI) versus fluoresencence 1 (FL1 for Annexin) plot. (**b**) Percentage distribution of viable cell associated with phosphatidylserine externalization of 48 h and 72 h capsaicin-treated and untreated (control) ORL-48 cells.

The cell death of untreated and capsaicin-treated ORL-48 cells was confirmed by measuring the phospholipid phosphatidylserine (PS) using FITC-Annexin V/PI. Treatment of ORL-48 cells with IC_50_ value of capsaicin for 48 h and 72 h induced apoptosis through the shift in cell population from early apoptosis (R5) to late apoptosis (R3) as shown in Figure 3a. As observed in this study, movement of capsaicin-treated ORL-48 cells through the quadrant stages showed a significant reduction in the percentage of viable cells, and a significant increase in the percentage of cells undergoing early apoptosis (R5) and late apoptosis (R3) following treatment with capsaicin for 48 h and 72 h. Furthermore, capsaicin-treated ORL-48 cells showed a faster movement through the quadrants from the viable stage (R4) to early apoptosis (R5) and then late apoptosis (R3) stage compared to untreated ORL-48 cells. The percentage of viable cells in untreated ORL-48 cells is around 84% compared to 72% and 61% in 48 h and 72 h capsaicin-treated ORL-48 cells, respectively. Thus, it was evidenced that capsaicin-treated ORL-48 cells decreased dramatically in comparison to untreated cells in a time-dependent manner (Figure 3b). In contrast, the percentage of early apoptosis (R5) and late apoptosis (R3) ORL-48 cells increased significantly, also in a time-dependent manner. This concedes with the chart that showed an 8% distribution of the viable cells for the untreated cells, but a vast elevation of 12% and 24% distribution in the 48 h and 72 h capsaicin-treated cells within the early apoptosis stage, respectively.

### 3.4. Determination of Caspase Activities and Disruption of Mitochondrial Membrane Potential

**Figure 4:**
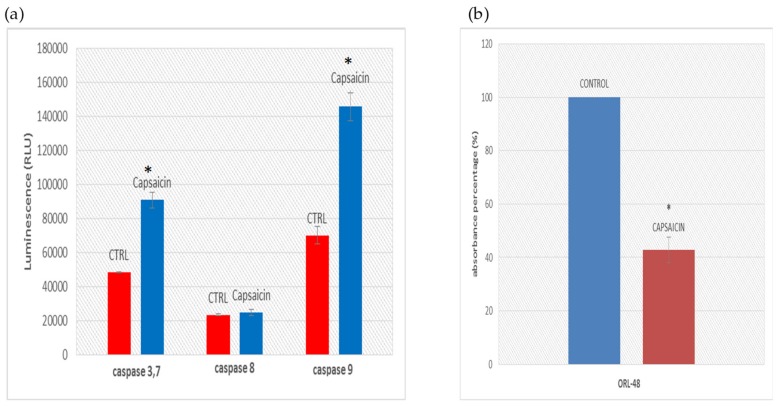
Determination of caspase activities and disruption of mitochondrial membrane potential of ORL-48 cells treated with capsaicin. (**a**) Caspase-3/7, -8 and -9 activities in 24 h capsaicin-treated and untreated (control) ORL-48 cells. Statistically significant differences between control and treated cells were set at * *p* < 0.05. (**b**) Absorbance percentage of TMRE-stained ORL-48 cells following 24 h exposure to capsaicin.

The caspases activity was quantified as Relative Luminescence Unit (RLU), where the luminescent intensity of caspase-3/7 and -9 activities in capsaicin treated cells increased significantly compared to untreated cells at *p* < 0.05. As shown in Figure 4a, the RLU value for caspase-8 activity was relatively low and not significant compared to the untreated cells (*p* > 0.05). The high RLU value observed in caspase-3/7 and -9 when compared to the control indicate a high possibility that the intrinsic (mitochondrial) pathway was involved for the apoptotic process of cell death of capsaicin-treated ORL-48 (Figure 4a). The changes in MMP (Δψ_m_) in TMRE-stained ORL-48 cells were measured by recording fluorescent intensity. The ORL-48 cells exhibited significantly (*p* < 0.05) lower fluorescence intensity of 42% absorbance after 24 h treatment with 200 μM capsaicin as compared to untreated cells of 100% absorbance and this could be due to membrane potential depolarization of the mitochondria (Figure 4b).

### 3.5. Cell Cycle Analysis

**Figure 5:**
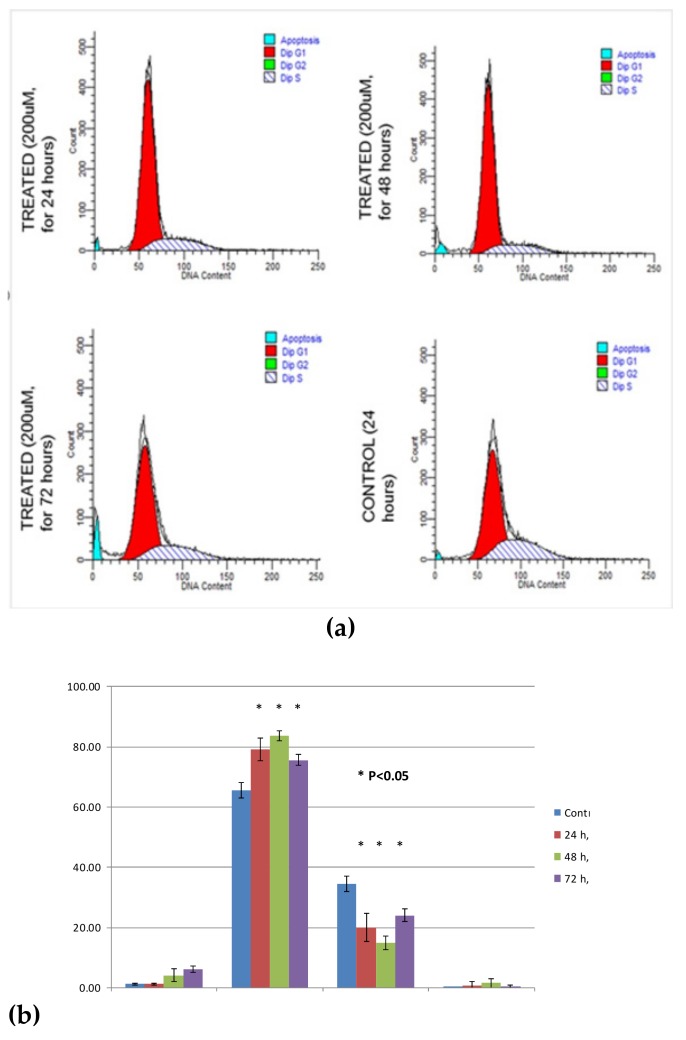
Cell cycle flow cytometric analysis of ORL-48 cells treated with capsaicin. (**a**) Cell density plot of apoptosis in ORL-48 cells treated with IC_50_ concentration of capsaicin for 24, 48 and 72 h. (**b**) Percentage of cell cycle distribution of untreated and capsaicin-treated ORL-48 cells (24, 48, 72 h) within each cell cycle phase.

The amplitude of the emitted fluorescence signals was measured and represented in a DNA histogram result of DNA distribution percentage and summarized in a plot as shown in Figure 5a. Figure 5a reveals the percentage for the cell cycle distribution for both capsaicin-treated and untreated ORL-48 cells that includes sub-G1 (apoptosis), G1-, S- and G2/M-phase. The percentage for the sub-G1 phase (apoptotic) for both the 24, 48, 72 h capsaicin-treated and untreated ORL-48 cells were 3%, 6%, 8% and 3% respectively. In the G1-phase, the percentage of the untreated cells was 65% before declining to a value of 36% within the S-phase. As for the 24 h capsaicin-treated ORL 48 cells, 79% of cell cycle distribution value can be observed within the G1-phase as compared to 20% value within the S-phase. More substantial decrease can be observed in 48h capsaicin-treated ORL-48 cells at 83% within the G1-phase to 16% S-phase. Subsequently, there is a decrease in the cell distribution of 72 h capsaicin-treated ORL-48 cells from 76% within the G1-phase to 23% within the S-phase. As for the G2-phase, cell cycle distribution of the 24 h capsaicin-treated ORL-48 was at 2%, increased within 48 h at 4% and decreased at its lowest within the next 72 h at 1%. This is then accompanied by the concomitant decrease in the percentage of capsaicin-treated ORL-48 cells in the S-phase as compared to the untreated cells (Figure 5b). These results indicate that the arrest of cell cycle of capsaicin-treated ORL-48 cells in the G1-phase is associated with the antiproliferative effect of capsaicin in a time-dependent manner.

## 4. Discussion

In this study, MTT Proliferation Assay was performed to assess cell viability and growth inhibition effect of capsaicin on ORL-48 cancer cell line. The assay involves cells uptake of MTT due to its plasma membrane potential and the net positive charge would be reduced by the formation of formazan by NAD(P)H-oxidoreductases. In order to ensure that no contact inhibition occurs when the cells achieve confluency, the cells were seeded sparse enough to allow linear growth. The cell inhibition effect of the ORL-48 cells was observed within 24, 48 and 72 h as capsaicin’s concentration increased. The results obtained from this study revealed that the IC_50_ value for capsaicin at 200 µM concentration was in line with most studies, as reviewed by Bley et al (2012) that showed capsaicin maximal effects reside within the lower concentration range, approximately 200–300 µM [17].

The growth inhibiting property of capsaicin towards ORL-48 oral cancer cell line as observed in our study has also been reported in other studies [25,26]. Apoptosis can be brought about by necrosis, a catastrophic (and easily discernible) type of cell death or apoptosis-type of cell death regulated by a series of signal cascades under a particular set of factors [27,28,29]. Apoptotic bodies, formed during the execution phase of the apoptotic process, have been described as progressive alteration of cell shape with deep cystoskeleton rearrangement due to cell membrane blebbing and this includes splitting of numerous cellular positions forming the pyknotic apoptotic bodies [30]. The exposed phosphatidyl-serine (PS) on the outer layer of the plasma membrane acts as a signal for recognition of phagocytes during the early stage of apoptosis [31]. Annexin V, a calcium-dependent protein used for characterization of apoptotic cell population in flow cytometry, works by binding to the exposed phosphatidyl-serine (PS) on the external layer of the membrane, with the aid of calcium ion [32]. It was observed in this study that the percentage of cells undergoing early apoptosis and late apoptosis increased significantly, thus confirming that apoptosis was the major mode of cell death induced by capsaicin in ORL-48 cells.

Morphological analysis for detection of apoptosis has been widely used and is based on certain distinctive features of the cells. Damaged cellular membranes will result in the release of cytosilic and organelle content into the surrounding environment, which will then allow propidium iodide to enter and bound to the concentrated bodies. Morphological observations of the ORL-48 cells when treated with capsaicin showed intensified green color of the cell nuclei when using the fluorescent microscope and this would further confirmed that apoptosis is the major mode of cell death in capsaicin treated ORL-48 cells. Generally, apoptosis by the intrinsic or mitochondrial pathway activates caspase-9 and extrinsic or death receptor pathway activates caspase-8 [33,34,35]. In this study, caspase-9 activity was significantly higher with the capsaicin-treated cells than the untreated cells. Permeability of the mitochondrial membrane is critical and plays a role in the regulation of apoptosis, in which the mitochondria act as the ‘bridge’ for signalling pathways [36]. Rapid increase in permeability of the mitochondrial membrane to solutes less than 1.5 kDa caused by charge difference between the matrix and cytosol is suggested as a major contribution to apoptosis, known as permeability transition. Release of cytochrome C and other apoptogenic mitochondrial proteins by various types of stimuli is critical for the mitochondrial pathway of apoptosis. The cytochrome C being released will bind to adaptor molecules, Apaf-1 and procaspase-9 leading to caspsase-9 activation resulting in the formation of multimeric apoptosme complex, which will subsequently activate caspase-3, -6 and -7 [36]. The results obtained in this study have also shown that the caspase-3/7 activity was significantly higher in capsaicin-treated ORL-48 cells thus implying that capsaicin induced the disruption of the mitochondria membrane in ORL-48 cells. According to our study, mitochondria membrane potential disruption of ORL-48 cells is significantly increased compared to the control cells, suggesting that the compound is critical in causing the release of apoptotic material from the cytoplasm. This is in agreement with a previous report where release of the apoptotic material from the cytoplasm has been shown to occur in the degradation phase of apoptosis [37].

Cell cycle is the main regulator for both cell population and growth. Thus, cell cycle has emerged as one of the desirable therapeutic targets when treating cancer. The cell cycle distribution was analyzed using flow cytometry, which in principle involves binding of the fluorescent stoichiometrically to the DNA and hence, the relative nuclear DNA distribution can be measured by the instrument [38]. The flow cytometry also enables a distinct identification of the four phases in cell cycle; G1- (apoptotic), G1-, S- (DNA synthesis), and G2/M- [39]. Several checkpoints in the cell cycle act as supervisory mechanisms and during DNA damage or cellular stress, it will trigger cell cycle arrest or apoptotic induction. We have shown in this study that the cell cycle was inhibited at the G1-phase. In the G1-phase, mRNA and proteins are being synthesized in preparation for the following phases of the cell cycle. Throughout the G1- stage, growth-dependent cyclin-dependent kinase (CDK) activity boosts DNA replication and initiates transition of the G1- to S- phase [40].We have also observed in this study a significant decline of cell population distribution percentage of the capsaicin-treated ORL-48 cells from the G1-phase to S-phase, confirming the antiproliferative effect of capsaicin. This finding is in line with a previous study where cell cycle arrest in the G1-phase has been reported in the human’s colon cancer by capsaicin [41].

Uncontrolled cell growth is an important phenomenon of cancer development and antitumor agent should possess the ability to prevent cancer cell growth. It is concluded from this study concluded that capsaicin possess a strong cytotoxic and antiproliferative property against ORL-48 cells, an Asian oral squamous cell carcinoma (OSCC) cell line by inducing apoptosis through mitochondria-mediated intrinsic pathway. The findings of this study would suggest that capsaicin has the potential to be developed as an antitumor agent for oral cancer therapy. 

## 5. Conclusions

In conclusion, the study demonstrated that capsaicin induced cytotoxic effect against an oral type of cancer cell line, ORL-48. In addition, capsaicin caused apoptosis through disruption of mitochondrial membrane potential via the intrinsic apoptotic pathway, resulting in cell cycle arrest.
